# Fractal dimension: a biomarker for detecting acute thromboembolic disease

**DOI:** 10.1186/cc11038

**Published:** 2012-03-20

**Authors:** K Hawkins, N Badiei, J Weisel, I Chernysh, PR Williams, MJ Lawrence, PA Evans

**Affiliations:** 1 Swansea University, Swansea, UK; 2 University of Pennsylvania, Philadelphia, PA, USA

## Introduction

This study investigates the potential use of rheometry to provide a structural biomarker for acute critical illness. Previous studies have reported an association of altered fibrin clot network architecture with several diseases including sepsis, bleeding or acute thromboembolic disease [1]. We investigate our biomarker by examining the relationship between thrombin generation and clot architecture in an *in vitro *model.

## Methods

Rheometry and confocal laser scanning microscopy (CLSM) were used to monitor and image the formation of fibrin clots. Clotting was initiated by the addition of different levels of thrombin to solutions of a fixed concentration of fibrinogen. Each sample was divided into two aliquots; one added to the measuring geometry of an AR-G2 rheometer and one to the microscope slide for the spinning disk CLSM (Olympus IX71).

## Results

**Figure 1:**
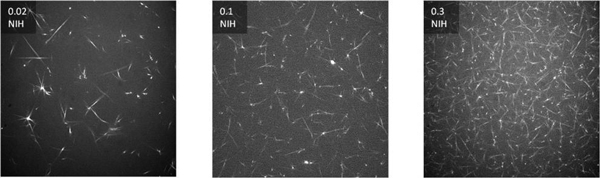
**CLSM micrographs of formed fi brin clots at thrombin levels of 0.02, 0.1 and 0.3 NIH**.

The micrographs of formed clots (Figure 1) show marked qualitative differences in clot architecture. Upon increasing the available thrombin, the clot network (visually) appears more dense. Table 1 shows the value of the structural biomarker, the fractal dimen-sion, that corresponds to the clots formed in Figure 1.

**Table 1:** Results of the fractal dimension obtained by rheometry of fibrin clots

Thrombin (NIH)	Fractal dimension
0.02	1.85
0.1	1.95
0.3	2.13

## Conclusion

We demonstrate, for the first time, that the fractal dimen-sion obtained by rheometry is a sensitive measure of visually observed structural differences within the fibrin network. Rheometrical detection of incipient clots formed in whole blood provides the clinician with a powerful tool for the diagnosis of thromboembolic disease.
